# Neurotoxicity of Tumor Immunotherapy: The Emergence of Clinical Attention

**DOI:** 10.1155/2022/4259205

**Published:** 2022-01-18

**Authors:** Benxia Zhang, Xue Li, Tao Yin, Diyuan Qin, Yue Chen, Qizhi Ma, Pei Shu, Yongsheng Wang

**Affiliations:** ^1^Clinical Trial Center, National Medical Products Administration Key Laboratory for Clinical Research and Evaluation of Innovative Drugs, West China Hospital, Sichuan University, Chengdu, Sichuan, China; ^2^Department of Thoracic Oncology, State Key Laboratory of Biotherapy and Cancer Center, West China Hospital, Sichuan University, Chengdu, Sichuan, China

## Abstract

Tumor immunotherapy brings substantial and long-term clinical benefits that can even cure tumors. However, the accumulation of evidence suggests that immunotherapy also induces severe and complex neurologic immune-related adverse events (ir-AEs) and even leads to immunotherapy-related death, which arouses the concern of clinicians. The timely and accurate identification of neurotoxicity helps clinicians detect and treat these complications early, thereby enhancing treatment efficiency and improving the prognosis of patients. At present, the mechanism of neurotoxicity caused by immunotherapy has not been completely elucidated. This paper mainly reviews the clinical features, pathogenesis, and therapeutic strategies of neurologic ir-AEs.

## 1. Introduction

Tumor immunotherapy refers to novel therapeutic measures that turn the immune system into a destructive force against tumors [1]. In general, tumor immunotherapy involves immune checkpoint inhibitors, immunostimulating antibodies, cytokine therapy, therapeutic cancer vaccines, adoptive cellular therapy, oncolytic immunotherapy, etc. [2]. In recent years, tumor immunotherapy has achieved significant success in various cancers and has been one of the hotspots in the life sciences, especially immune checkpoint inhibitors and adoptive cellular therapy [3]. Immune checkpoint inhibitors (ICIs) are antibodies that target crucial signaling pathways, such as programmed death 1 (PD-1)/programmed death-ligand 1 (PD-L1), and cytotoxic T-lymphocyte-associated protein 4 (CTLA-4), to improve the activation of T cells and enhance the immune response to cancer cells. Pembrolizumab (Keytruda), a humanized monoclonal anti-PD-1 antibody, is the first anti-PD-1 antibody approved by the FDA for the treatment of patients with unresectable or metastatic melanoma, non-small-cell lung cancer (NSCLC), head and neck squamous cell carcinoma, cervical cancer, colorectal cancer, and gastric/gastroesophageal junction cancer [4–10]. Adoptive cellular therapy transmits the sensitized T cells to patients with low cellular immune function, which helps patients obtain antitumor immunity. Adoptive cellular therapy, especially chimeric antigen receptor (CAR) T cell therapy, has gained unprecedented success among hematologic tumors [11]. Kymriah is the first CAR-T therapy based on CD19 approved by the FDA in 2017 [12]. Related studies have reported that the complete response rate of CD19-CAR-T cells in hematological malignancies is approximately 88–90% [13, 14].

Life sciences professionals believe that immunotherapy will revolutionize cancer treatment. Nevertheless, side effects caused by immunotherapy should not be underestimated, such as skin damage, ir-AEs, bone marrow depression, tumor lysis syndrome, coagulation disorders, etc. [15–18]. Recently, with the widespread use of immunotherapy, neurologic ir-AEs, such as encephalopathy, meningitis, neuropathy, and seizures, have aroused the concern of clinicians. It is a dose-limited factor of cancer immunotherapy and affects the prognosis and quality of life [15]. The incidence of the neurologic ir-AEs has varied in different immunotherapeutic agents. Blinatumomab is a bispecific T-cell engager (BiTE) targeting CD19 on malignant B cells and CD3 on normal host T cells. In a multicenter, single-arm, open-label phase 2 study on patients with relapsed or refractory B-precursor acute lymphoblastic leukemia, the distribution of any grade neurologic ir-AEs frequency was 52% among patients receiving blinatumomab, and grade 3 or 4 neurologic ir-AEs occurred in 11% and 2% patients, respectively [19]. The incidence rate for any grade ICIs-related neurologic ir-AEs in patients included in clinical trials has been reported to be approximately 3.8% to 12% [20]. The distribution of grade 3-4 neurologic ir-AEs frequency was 0.7% among patients receiving anti-CTLA-4 (ipilimumab and tremelimumab), 0.4% among patients receiving anti-PD1 (nivolumab, pembrolizumab, lambrolizumab, and pidilizumab), and 0.7% among patients receiving a combination therapy (anti-CTLA-4 + anti-PD1) [20]. Any grade CAR-T-related neurologic ir-AEs, by contrast, have the highest incidence. 62.3% of patients experienced any grade neurologic ir-AEs [21]. Notably, in the JCAR015 Phase 2 Clinical Trial (ROCKET) conducted by Juno Therapeutics, five patients died because of severe cerebral edema, causing the cessation of clinical trials by the FDA [22]. Emphasizing the importance of recognizing the neurologic ir-AEs related to immunotherapy early, especially CAR-T therapy, can help clinicians differentiate treatment-related adverse effects from the progression of cancer within the nervous system and how intervening sooner leads to better outcomes. In this review, we shed new light on the clinical characteristics and possible mechanisms of neurologic ir-AEs and highlight their clinical management.

## 2. Neurotoxic Features of Immunotherapy

Neurologic ir-AEs are typically characterized by toxic encephalopathy manifesting as headache, delirium, confusion, dysphasia, impaired fine motor skills, somnolence, etc. [23]. Moreover, rare complications, such as Guillain–Barre syndrome (GBS) [24–27], myasthenia gravis (MG) [28, 29], cerebellar dysfunction, and aseptic meningitis have also been reported by several clinical cases. Most of these adverse effects are grade 1 or 2, but in the clinical treatment process, ≥grade 3 neurologic ir-AEs also occur in patients (the severity of ir-AEs is graded based on the Common Terminology Criteria for Adverse Events version 5.0 [30]). Clinicians need to master the neurologic ir-AEs characteristics. Here, we summarize the performance of neurologic ir-AEs, hoping to help clinicians make a rapid diagnosis.

### 2.1. Headache

Headache is one of the most common unspecific manifestations of neurologic ir-AEs associated with immunotherapy. Patients with a history of headache before treatment may be more susceptible to headache following treatment [31–34]. Patients may suffer from headaches during or shortly after the first infusion of cetuximab [33, 35], bevacizumab [36], ipilimumab [32], rituximab [37], chimeric antigen receptor T cells (CAR-T) [21], and pembrolizumab [38] (Table 1). The symptoms are mostly self-limited, persisting for several days and decreasing soon after finishing the treatment [18, 39]. In addition, a headache might also be a manifestation of other complications, such as aseptic meningitis and acute encephalopathy.

### 2.2. Seizures

Seizures occur because of acute, recurrent, paroxysmal brain disorders caused by the excessive firing of brain neurons, and they may be related to immune system activation with a significant accumulation of inflammatory cytokines [40–42]. Rituximab, an anti-CD20 chimeric monoclonal antibody, is generally well tolerated in patients with relapsed or refractory non-Hodgkin's lymphoma [43]. However, rituximab-induced seizures were occasionally reported and usually appeared within hours after injection of rituximab [44]. Seizures are the most common presenting symptom of posterior reversible encephalopathy syndrome (PRES) induced by rituximab [44–47]. PRES is thought to be associated with the failure of the autoregulation of cerebral blood pressure and local central nervous system inflammation. Once the patients develop seizures, rituximab infusion should be interrupted [48]. Other immunotherapeutic agents, such as bevacizumab, ipilimumab, and sunitinib, may also induce PRES in less than 1% of the treated patients [49–51]. In addition, blinatumomab causes seizures in 3% to 4.8% of the treated patients in clinical studies, and the seizures begin within the first two days of infusion [34, 52]. Zimmer, L. and colleagues have found that 0.4% of patients receiving pembrolizumab or nivolumab developed seizures in a retrospective study of 496 patients with melanoma. Additionally, seizures occur upward in 3% of patients after CD19-CAR T cells infusion and are related to the dose of CAR-T cells. Seventy-two percent of patients with severe neurologic ir-AEs during CAR-T treatment might develop generalized tonic-clonic seizures [21]. Patients who suffered from seizures during the treatment can be evaluated with head computed tomography (CT), magnetic resonance imaging (MRI), electroencephalograms (EEGs), and lumbar punctures. In general, the EEG findings were frontal intermittent rhythmic delta activity (FIRDA) and a diffused or frontal slowing wave [21]. The analysis of cerebrospinal fluid (CSF) collected by lumbar puncture may indicate lymphocytosis [13]. CT and MRI were used to exclude differential diagnoses, such as tumor metastasis. Seizures usually improved with drug withdrawal, but steroids may be required to reverse in some circumstances [18, 53].

### 2.3. Encephalopathy

Acute encephalopathy is characterized by acute alteration of mental status, which is a common immunotherapy-related neurotoxic symptom [21, 54, 55]. Clinical features included confusion, delirium, cognitive impairment, speech disorders, impaired attention, and dizziness [55, 56]. Immunotherapeutic agents, such as ipilimumab, blinatumomab, bevacizumab, and CAR-T cells therapy, might induce acute alteration of mental status in treated patients [13, 50, 55, 57, 58]. In a study of adult patients with relapsed or refractory CD19^+^ B acute lymphoblastic leukemia, grade ≥3 neurotoxicity occurred in 50% of patients either concurrent with or after cytokine release syndrome (CRS), and most of them manifested as mild-to-severe encephalopathy, focal neurologic deficits, and seizures [59]. Although the pathogenesis of CAR-T cell therapy-induced encephalopathy remains inadequately understood, it is believed that endothelial activation, capillary leakage, disseminated intravascular coagulation, and increased blood-brain barrier (BBB) permeability are related to CAR-T cell induced neurotoxicity [42]. Similar T cell-mediated endothelial activation and the disruption of BBB are observed with blinatumomab [41]. CT, MRI, and lumbar punctures can be performed to exclude brain metastasis. In recent years, studies have discovered that serum IL-6, IL-8, IL-10, IL-15, CCL2, TGF-*β*, TNF-*α*, C-reactive protein (CRP), and IFN-*γ* concentrations early after CAR-T cell infusion may be helpful to identify whether patients are at risk of developing life-threatening toxicity [59, 60]. Blinatumomab and CAR-T therapy-related encephalopathy are primarily reversible and can be resolved several days without intervention or by discontinuation of the drugs [13, 42, 52].

Chronic encephalopathy, such as leukoencephalopathy, could be induced by direct brain parenchyma damage. Chronic encephalopathy usually develops months after receiving cancer immunotherapy [50]. Clinical symptoms included progressive dementia, cognitive dysfunction, personality change, blunted effect, and amnestic syndrome. A specific cause of chronic mental state changes is progressive multifocal leukoencephalopathy (PML), a rare demyelinating disease induced by the activation of the latent John Cunningham (JC) virus. PML primarily occurs in individuals with severe immunosuppression, especially with HIV-1 infection [61]. Recently, PML has been increasingly diagnosed in patients receiving immunotherapeutic agents that modulate immune system functions, such as rituximab [62], brentuximab [63], nivolumab [64], alemtuzumab [65], and CAR-T therapy [66]. Related research has indicated that cancer immunotherapy-induced PML might rely on activating previously exhausted polyoma-specific T cells [64–66]. Diagnostic assessment should be comprehensive, which includes neuropsychological testing, lumbar puncture, MRI, and laboratory assessments. Brain atrophy and ventriculomegaly may be seen in MRI imaging for some patients with chronic encephalopathy [63, 65, 66]. Interventions include the discontinuation of treatment, plasmapheresis, and steroids.

### 2.4. Peripheral Neuropathy

Peripheral neuropathy includes peripheral sensory neuropathy and peripheral motor neuropathy. Peripheral neuropathy is a dose-limiting neurotological adverse effect secondary to tumor immunotherapy. Most of the peripheral neurological symptoms involve sensory nerves and are almost grade 1 or 2 [67]. Most peripheral neuropathies present with symmetric and “stocking- and glove-like” paresthesia. Brentuximab, an antibody against CD30 on the B and T cells, is used to treat an anaplastic large cell and refractory Hodgkin lymphoma. Peripheral neuropathy developed in 22% of patients receiving brentuximab and typically presented with grade 1 or 2. The median time to the onset of peripheral neuropathy was nine weeks (range from 3 to 24 weeks) [68]. Brentuximab should be stopped once diagnosed with peripheral neuropathy until symptoms improve to grade 1. After resolution, brentuximab could be reinitiated at a lower dose. Additionally, peripheral sensory neuropathy is also a prominent side effect of ipilimumab [55], nivolumab [69], pembrolizumab [70], and rituximab [48]. The possible mechanism is that the drugs disturb the normal axonal transport [48, 67, 68, 71–77]. For patients with peripheral neuropathy, CT, biochemical tests, and MRI can be helpful for diagnosis. CT and MRI are used to exclude brain metastasis, while the biochemical examination of CSF may show only a protein increase [78]. Peripheral neuropathy is often self-limited and can be resolved by drug withdrawal.

### 2.5. Cerebellar Dysfunction

The main functions of the cerebellum are maintaining the balance of the human body and controlling the posture and gait. Cerebellar dysfunction presents with ataxia, nystagmus, confusion, scanning speech, and confusion. Cerebellar dysfunction is a rare complication of ipilimumab [79, 80], trastuzumab [81], and blinatumomab [53]. It is usually pronounced in elderly patients and patients with preexisting neurological conditions or renal dysfunction. Evidence shows that 2.1% of patients using blinatumomab may develop cerebellar dysfunction, and the symptoms are usually reversible with the discontinuation of the drug [82]. CT and MRI could be used to exclude brain metastasis, and EEG may be observed only as a slow wave.

### 2.6. Myasthenia Gravis (MG)

MG, an autoimmune disease caused by the dysfunction of the neuromuscular junction, is a rare but life-threatening side effect related to tumor immunotherapy. MG usually involves the extraocular muscles at the beginning, followed by other muscle groups. The clinical symptoms include fatigable weakness in proximal limb muscles, ptosis, dyspnea, and diplopia [83]. In severe cases, patients involving respiratory muscle dysfunction may need breathing support. Immunotherapy-induced MG is an increasingly recognized and feared complication of ICIs [84, 85]. Although MG exacerbation has been reported in patients who have had stable myasthenia during ICIs therapy, ICIs-related MG appears to be primarily new with no previous clinical history of MG [29, 86]. The average onset of MG-related symptoms was four weeks (from six days to 16 weeks) of ICIs. The median time from symptom onset to respiratory failure demanding intubation was seven days [83]. Almost all patients with ICIs-related MG need hospitalization, and 40–50% of them require mechanical ventilation [83, 87]. The elevation of acetylcholine receptor (AChR) antibodies was reported in laboratory tests in 66% of the tested patients [83]. For seronegative patients for AChR antibodies, 50% to 70% were seropositive for antibodies to muscle-specific kinase (MUSK). Electromyography could detect the features of MG in 41% of patients. CT and MRI were usually normal. The discontinuation or withholding of ICIs was recommended. According to the severity of the condition, intravenous methylprednisolone, intravenous immunoglobulin, or plasmapheresis can be used to improve symptoms [85, 88]. A few cases of MG have also been reported in studies combining anti-PD-1/PD-L1 monoclonal antibody with anti-CTLA-4 monoclonal antibody [89], but which one induced MG remains to be confirmed. We found that most patients with MG are women. Whether this is a coincidence or drug-induced MG affects females remains to be clarified.

### 2.7. Guillain–Barre Syndrome (GBS)

GBS is an autoimmune peripheral neuropathy characterized by the demyelination of peripheral nerves and nerve roots, infiltration of small vascular inflammatory cells, and acute inflammatory demyelinating polyneuropathy (AIDP) [90]. Episodes are triggered mainly by an infectious process. Clinical manifestations include progressive ascending symmetry paralysis, proximal limb weakness, and peripheral sensory disturbance. ICIs-related GBS is a rare neurologic ir-AE with an incidence of less than 1% [25, 26], and it frequently presents as swiftly ascending paresthesia in the hands and feet over days within the first 3-4 ICIs cycles. Increased creatine kinase (CK) ranging from mild to high levels presents more frequently in ICIs-related MG than ICIs naive patients [91]. TCR-T cell therapy-induced GBS has also been increasingly recognized. The onset of GBS-related symptoms ranges from 19 days to 142 days of TCR-T therapy initiation and shows a rapidly progressive and bilateral ascending weakness [24, 92]. The diagnosis is mainly clinical, but may be facilitated by additional investigations, such as brain MRI, electromyography (EMG), nerve conduction studies, and lumbar puncture. MRI of the brain and the spinal cord can be used to exclude disease progression. EMG may show a diagnostic significance for generalized motor and sensory demyelinating polyneuropathy. Albuminocytologic dissociation is often noted in CSF, but it is worth noting that there have been cases of rapidly ascending weakness that lack classic albuminocytologic dissociation. Although high-dose corticosteroid therapy (40 mg methylprednisolone administered by intravascular injection twice per day) has been used to improve the symptoms in cases, corticosteroids are of little benefit in immunotherapy-induced GBS, and the current guidelines recommend consideration of corticosteroid therapy in combination with IVIG or plasmapheresis [25].

### 2.8. Aseptic Meningitis

Aseptic meningitis has been noted in several different immunotherapeutic agents, such as cetuximab [93], pembrolizumab, nivolumab [94], and ipilimumab [95]. Meningitis was more likely to present with additional non-neurologic immune-related adverse effects. Symptoms include unspecific headaches, photophobia, neck stiffness, and altered mental status. It is challenging to distinguish meningitis from encephalitis in patients with metastatic cancer or patients presenting with new seizures and cognitive impairment. Aseptic meningitis was probably related to markedly elevated inflammatory cytokines, such as IL-6, IL-2, interferon-*γ*, and tumor necrosis factor-alpha (TNF-*α*). Patients who developed aseptic meningitis can be tested by lumbar punctures, CT, and contrast brain MRI. CSF findings typically demonstrate lymphocytes as the central part, normal-to-low glucose values, and elevate protein. MRI might show abnormal diffuse thickening of the dura mater. CT, bacterial culture, viral culture, and fungal culture are often negative. Steroids can be used to improve the symptoms. NCCN guidelines recommend patients who experience mild-to moderate-grade meningitis rechallenge treatments when there is complete symptom resolution [96].

Other symptoms, including transient vision loss, hypothalamic or pituitary gland disorder [97, 98], depression, dizziness [99, 100], acute inflammatory demyelinating polyneuropathy, and chronic inflammatory demyelinating polyneuropathy (CIDP), were also occasionally reported. According to the clinical symptoms, blood tests, biochemical tests, cerebrospinal fluid puncture, and head imaging can be used to make a precise diagnosis, followed by symptomatic supportive care.

### 2.9. Predictors and Assessment of Neurologic ir-AEs

At present, there are still no proven biomarkers to predict neurologic ir-AEs in patients undergoing the administration of ICIs. However, limited evidence has indicated that CAR-T-related neurotoxicity is related to tumor burden, the number of CAR-T cells infused, previous lymphodepleting chemotherapy (fludarabine and cyclophosphamide), age, and previous chemotherapy. Neurologic ir-AEs can be defined and graded using the CTCAE or ASTCT ICANS grading system. Indeed, recent studies have proved that the CTCAE grading system overestimates the incidence of grade 1 or 2 neurological adverse events [101]. Guidelines for immune-related adverse effect management have been established by the American Society of Clinical Oncology (ASCO) and the National Comprehensive Cancer Network (NCCN).

### 2.10. Pathogenesis of Neurologic ir-AEs

Traditional chemotherapy- and radiotherapy-induced neurotoxicity has been widely recognized in patients with cancer. The central nervous system contains stem cells and slowly splitting glial cells that supply some neuronal populations. Chemotherapy preferentially acts on the dividing cells. The central nervous system is an unexpected target of agents that affect the dividing cells. Likewise, radiation therapy (RT) targets the dividing cells and can cause damage to the neural structures directly or indirectly by causing vascular damage, fibrosis of neural structures, and endocrine disturbance. Accordingly, depending on the region of the brain involved, RT can lead to stroke-like migraine, aphasia, hemiparesis, seizures, and so on.

Compared with traditional therapy, neurologic ir-AEs are more severe. Immunotherapeutic agents can activate the endothelial cells and damage the nerve system directly by upregulating the immune system to release cytokines. In some cases, these cytokines can cause brain edema, resulting in the death of patients [16, 102]. Given the protection of BBB and the low proliferative rate of neurons, the nervous system should be well protected from the toxic effects of immunotherapy and traditional therapy. However, some immunotherapeutic agents can still concentrate in the cerebrospinal fluid to act on the normal brain tissue [103]. Additionally, preexisting neurologic comorbidities are correlated with an increased risk of neurologic ir-AEs. Moreover, cyclophosphamide and fludarabine lymphodepletion, a higher infused CAR-T cell dose, and a higher burden of malignant cells increased the risk of developing CAR-T therapy-related neurotoxicity [42].

### 2.11. Upregulation of the Immune System

Immunotherapeutic agents such as blinatumomab and CAR-T cells can activate the T lymphocytes to release various cytokines into the blood and cause a group of symptoms. This process is called CRS [104, 105]. Although recent research shows that the neurologic ir-AEs are irrelevant to CRS, there is evidence that patients who undergo CRS after immunotherapy are more vulnerable to developing neurologic ir-AEs [13, 106]. Studies have demonstrated that neurologic ir-AEs secondary to CAR-T therapy is probably related to the markedly elevated inflammatory cytokines, such as IL-6, IL-2, interferon-*γ*, and TNF-*α* [16, 102, 107, 108]. Moreover, Norelli and colleagues proved that elevated IL-1 and IL-6 after CAR-T cell infusion are secreted by human monocytes [109]. In addition, aseptic meningitis is related to high levels of IL-6, IL-2, and interferon-*γ* in meninges. Seizures, acute encephalopathy, and peripheral neuropathy secondary to immunotherapy are also associated with high levels of cytokines [59, 60, 110, 111]. Importantly, immunotherapeutic agents-induced high levels of cytokines in the cerebrospinal fluid can lead to cerebral edema [112], which can cause paralysis, motor aphasia, and even the death of patients. Mayhan, W. G. found that TNF-*α* can increase the permeability of BBB and some peripheral organ barriers, allowing the kinds of cytokines to enter the central nervous system [113]. Therefore, TNF-*α* might play an important role in the pathology of immunotherapy-induced neurotoxicity.

Moreover, Hua-bing Wang et al. demonstrated that anti-CTLA-4 antibody treatment provoked MG by enhancing the T cell responses to AChR and increasing anti-AChR Ab production in mice [28]. Nivolumab-induced MG has also been found to be associated with increased anti-AchR antibodies [85]. In addition, some immunotherapeutic agents can activate the immune system to produce more cytotoxic T cells and subdue the regulatory T cells, leading to decreased peripheral tolerance to autoantigens, resulting in autoimmunity in different organs and causing symptoms such as GBS [25, 114, 115].

### 2.12. Cross-Reactivity with Cells from Normal Tissue

Cross-reactivity between the cells from the nervous system and tumor cells contributes to developing immunotherapeutic neurological toxicity [103]. “On tumor off target” adverse effect is defined as some target drugs binding to antigens expressed in the brain tissue that are similar to the antigens expressed in tumors. It has been confirmed that the melanocytes and Schwann cells are derived from the same neural crest and share some similar antigens. Therefore, an immune response against the melanocyte antigens may also attack similar antigens on the Schwann cells. Wilgenhof, S. and colleagues demonstrated that ipilimumab-induced GBS is caused by an incorrect attack on the cell surface ganglioside-related epitopes expressed in the human peripheral nerve axolemma [25]. Immunotherapy-induced chronic encephalopathy and headache were also related to the “on tumor off target” effect [116]. However, it is regrettable that the “on tumor off target” effect is extremely difficult to predict from preclinical studies because of the difference in the antigenic repertoire between the animal models and humans.

Moreover, some specific antigens are expressed not only in the tumor but also in the normal tissue. Immunotherapy targeting these same antigens in the normal tissue may cause side effects. In a clinical trial, researchers found that CD19 was regionally or temporally expressed on the neurons or glial cells. Therefore, the infused CD19 CAR-T cells can attack these neurons or glial cells [117]. In addition, the inhibitors of VEGF can increase the risk of stroke, ischemia of the nerve, and temporal arteritis in patients by increasing the permeability of the blood vessels and inducing thrombosis in newly formed tumor-associated blood vessels and normal vessels [118]. These processes might lead to hemiplegia, numbness, transient vision loss, etc., in patients receiving anti-VEGF antibodies [119–121]. The previous studies of CD22 have confirmed that CD22 is highly expressed on aged microglia [122]. However, CD22-CAR-T therapy-related neurologic ir-AEs have not been reported to have a higher incidence.

### 2.13. Direct Injury

A few immunotherapeutic agents directly penetrate BBB and cause short-term or long-term injury to the central nervous system progenitor cells and nondividing oligodendrocytes. Gust, J. et al. have discovered that severe neurotoxicity is associated with disseminated intravascular coagulation (DIC), elevated prothrombin time, and higher peak concentrations of CRP, ferritin, and multiple cytokines, including IL6, IFN-*γ*, and TNF-*α*, after CAR-T cells infusion. Furthermore, they demonstrated that the pericytes secreted more IL6 and VEGF via IFN-*γ* and TNF-*α*. Both IL6 and VEGF activate the endothelial cells and increase BBB permeability. In addition, IFN-*γ* also downregulates PDGFR*β* and upregulates the cleaved caspase-3 expression, consistent with the induction of pericyte stress. The angiopoietin (ANG)–TIE2 axis, a pathway that regulates the endothelial cell quiescence and activation, has been described to be abnormally activated in patients with grade ≥4 neurotoxicity compared to those with grade ≤3 neurotoxicity. These findings indicated that increased BBB permeability allowed the high concentrations of cytokines such as IFN*γ* and TNF*α*, to transit into CSF, which resulted in pericyte stress and secretion of cytokines that promoted a further increase of BBB permeability [42].

### 2.14. Others

In addition, scientists have found that the existing hyperferritinemia in patients with neurologic ir-AEs raises the probability that the activation of macrophage-lineage cells may be associated with the pathogenesis of neurologic ir-AEs [123]. Autoimmune hypophysitis might cause hormonal deficiencies, which will lead to the changes of the neurotransmitter in the synaptic cleft [124]. It has also been reported that the activation of voltage-gated sodium channels can lead to axonal injury, and the gain-of-function mutations of the sodium channel may be linked to peripheral neuropathy [125].

### 2.15. Treatments

The exact mechanism of neurologic ir-AEs remains to be elucidated. Most data are obtained from anecdotal reports and case series. Generally, the management aims to prevent further clinical deterioration and avoid the manifestation of irreversible neurological deficits. Close monitoring, drug dose adjustment, or withdrawal might be effective for patients with grade 1 or 2 neurotoxic adverse effects [74, 106]. Immunotherapeutic agents should be discontinued in patients who develop grade ≥3 neurotoxicity, and corticosteroids are generally used at most centers.

Immunotherapy-induced aseptic meningitis is generally self-limited. In severe cases, patients can be treated with intravenous high-dose methylprednisolone (about 80 mg–160 mg), dexchlorpheniramine, or equivalent metered dexamethasone [95, 111, 126]. Dexchlorpheniramine 50 mg can be applied before treatment to prevent the occurrence of aseptic meningitis [127]. For patients who develop seizures or GBS during the administration of immunotherapy, the therapeutic agents should be discontinued immediately, and glucocorticoids are needed for management as soon as possible [26, 128, 129]. High-dose glucocorticoids may be required if the symptoms further deteriorate [25, 130, 131]. Peripheral neuropathy induced by immunotherapy can also be treated with glucocorticoids. For severe peripheral neuropathy involving limb motor dysfunction, hormone shock therapy (methylprednisolone 7.5–30 mg/kg/day) can be used. If symptoms worsen, the addition of tacrolimus 0.15 mg/kg twice a day to hormone shock therapy significantly improved the prognosis of patients [78]. In addition, high-dose corticosteroids and hormone replacement are also supposed to be used for patients with therapy-induced hypophysitis [132–135]. An elaborative approach to the glucocorticoid taper and appropriate patient counseling is necessary to assure a successful taper after the neurological symptoms have abated.

Immunoglobulin is another commonly used drug. For patients with immunotherapy-induced MG or GMS, immunotherapeutic agents should be discontinued immediately, and intravenous immunoglobulin can relieve symptoms [25, 26]. Patients who experience mild to moderate grade immunotherapy-induced neurologic ir-AEs can rechallenge treatments upon complete symptom resolution. However, if the immunotherapy-induced neurologic ir-AEs occur more than once, the agents should be permanently discontinued.

Sedative drugs and antiepileptic drugs have been proposed to address immunotherapy-induced seizures. In general, intravenous lorazepam or oral levetiracetam can be used to improve the symptoms of seizures [26, 128, 129, 131]. Additionally, antidepressants can also be used to control peripheral neuropathy in some cases [136–138].

Anti-IL-6 receptor antibodies, such as tocilizumab can be used to treat CRS. Fortunately, tocilizumab mainly acts on monocytes and macrophages so that the efficacy of immunotherapeutic agents will not be significantly weakened [107, 117]. However, tocilizumab has no effect in most cases of neurologic ir-AEs. It may be because of the pathological differences between CRS and neurologic ir-AEs and poor penetration of tocilizumab across BBB. Recently, Norelli and colleagues have found that IL-1 receptor antagonists may be helpful for controlling the symptoms of CAR-T-related neurotoxicity.

In addition, plasmapheresis can also help improve the symptoms of peripheral neuropathy and drug-induced MG [26, 78]. Moreover, other cytokine inhibitors, such as itacitinib and ibrutinib, have been shown to reduce CAR-T cell-related toxicity in preclinical models [139, 140].

## 3. Conclusion

With the rapid development and widespread use of immunotherapy, its therapeutic effect has been widely recognized by clinicians. However, increasing attention has also been paid to immunotherapy-induced adverse effects simultaneously, especially neurotoxicity, because it is a limiting factor for accomplishing the course of treatment. Compared with traditional radiotherapy and chemotherapy, neurologic ir-AEs secondary to immunotherapy are more complex because of some new patterns of neurotoxic symptoms. We are now facing the following problems: firstly, the mechanisms of neurologic ir-AEs have not yet been elucidated. Secondly, there are still no specific diagnostic criteria for neurologic ir-AEs. Finally, there is no consensus on how to address these complications. Thus, a large number of studies concerning the questions mentioned above are supposed to be carried out. Meanwhile, the existing treatment strategies should be optimized to decrease the risk of nervous system damage and improve the survival of patients.

## Figures and Tables

**Table 1:** Clinical features of immunotherapy-induced neurologic ir-AEs.

	Agents	Clinical features	Diagnostic assessments	Treatments
Headache	Cetuximab; rituximab (rituxan); lucatumumab; ipilimumab; pembrolizumab; tremelimumab	Unspecific	The status of patients should be monitored closely	Mostly self-limited
Acute encephalopathy	Rituximab; ipilimumab; bevacizumab; alemtuzumab; tremelimumab; pembrolizumab; blinatumomab; CD19-CAR-T	Confusion, delirium, cognitive impairment, speech disorders, impaired attention, and dizziness	MRI, CT, EEG, and lumbar punctures	Drug pausing or withdrawal; steroids
Chronic encephalopathy	Rituximab; brentuximab; nivolumab; alemtuzumab; CAR-T therapy	Progressive dementia, cognitive dysfunction, personality change, blunted effect, and amnestic syndrome	Neuropsychological testing, lumbar puncture, MRI, EEG, and laboratory assessments	Discontinuation of the treatment, plasmapheresis, and steroids.
PRES	Rituximab; blinatumomab; CD19-CAR-T; bevacizumab; ipilimumab; sunitinib	Seizures, headache, confusion, visual changes	MRI, CT, lumbar punctures, EEG, and blood pressure assessment	Discontinue immunotherapy, prevent ischemia, steroids, and antiepileptic drugs
Aseptic meningitis	Cetuximab; pembrolizumab; nivolumab; ipilimumab; CD19-CAR-T	Unspecific headaches, photophobia, neck stiffness, and altered mental status	Lumbar punctures, CT, MRI bacterial culture, viral culture, and fungal culture	Generally self-limited. Drug withdrawal, intravenous high-dose methylprednisolone, dexchlorpheniramine, or equivalent metered dexamethasone
Peripheral neuropathy	Rituximab; ofatumumab; dacetuzumab; brentuximab vedotin; gemtuzumab ozogamicin; ipilimumab; pembrolizumab; nivolumab; blinatumomab; provenge vaccine	Sensory deficits, motor deficits	CT, MRI, lumbar punctures, electrophysiological assessment, and clinical assessment	Drug withdrawal and glucocorticoid; severe peripheral neuropathy: methylprednisolone 7.5–30 mg/kg/day plus tacrolimus 0.15 mg/kg twice a day, plasmapheresis
Cerebellar dysfunction	Ofatumumab; trastuzumab; ipilimumab; blinatumomab	Ataxia, nystagmus, confusion, scanning speech, and confusion	MRI, CT, EEG	Drug withdrawal and steroids
MG	Ipilimumab; pembrolizumab	Fatigable weakness in proximal limb muscles, ptosis, dyspnea, and diplopia	Blood test for AChR antibodies and MUSK, electromyography, CT, and MRI	Discontinuation or withholding of drugs was recommended; corticosteroids and immunoglobulin
GBS	ICIs; TCR-T therapy	Progressive ascending symmetry paralysis, proximal limb weakness, and peripheral sensory disturbance	Clinical features, MRI, electromyography, nerve conduction studies, and lumbar puncture	Discontinue immunotherapy, corticosteroids therapy combination with IVIG or plasmapheresis

PRES: posterior reversible encephalopathy syndrome; GBS: Guillain–Barre syndrome; MG: myasthenia gravis; ICIs: immune checkpoints inhibitors.

## Data Availability

The data used to support the findings of this study are included within the article.
